# A Case of Duodenal Adenocarcinoma Effectively Highlighted by Linked Color Imaging and Confirmed by Histopathology

**DOI:** 10.7759/cureus.53582

**Published:** 2024-02-04

**Authors:** Kimitoshi Kubo, Xinhan Zhang, Ikko Tanaka, Noriko Kimura

**Affiliations:** 1 Department of Gastroenterology, National Hospital Organization Hakodate National Hospital, Hakodate, JPN; 2 Department of Pathology, National Hospital Organization Hakodate National Hospital, Hakodate, JPN

**Keywords:** carbonic anhydrase ix, linked color imaging, differential diagnosis, duodenal adenoma, duodenal adenocarcinoma

## Abstract

While the differential diagnosis of duodenal adenocarcinoma versus adenoma remains the key to determining treatment strategies in patients with suspected duodenal adenocarcinoma, the role of linked color imaging (LCI) in their differential diagnosis remains insufficiently documented. In this case, esophagogastroduodenoscopy (EGD) was performed on a 67-year-old man for anemia, which revealed a 20-mm-sized, whitish, partially reddish, pedunculated lesion located in the duodenal bulb on white light imaging. Using LCI, the lesion was highlighted as a whitish, pedunculated lesion with its central and inferior areas depicted as orangish and reddish, respectively. Endoscopic mucosal resection was performed on the suspicion of an adenocarcinoma for biopsy and endoscopic diagnosis. Histological examination revealed the lesion to be an adenocarcinoma contained in an adenoma: papillary, type 0-Ip, measuring 20x20 mm, pTis (M), involving no lymphovascular invasion. This case appears to underpin the usefulness of LCI in the differential diagnosis of duodenal adenocarcinoma.

## Introduction

Duodenal adenocarcinoma is rare, accounting for about 20-25% of all cancers of the small intestine or 5% of all gastrointestinal tumors [1]. While the differential diagnosis of duodenal adenocarcinoma versus adenoma remains the key to determining treatment strategies in patients with suspected duodenal adenocarcinoma, it poses challenges, with the accuracy of its preoperative biopsy-based diagnosis reported to be 68-71.6% and with the role of endoscopy in its differential diagnosis shown to be inconclusive [2,3].

Recently developed for advanced image-enhanced endoscopy procedures, linked color imaging (LCI) (Fujifilm Holdings Corporation, Tokyo, Japan) serves to highlight slight differences in color between mucosal surface structures and vessels, likely reflecting differences in their reflectance at short wavelengths, thus allowing them to be easily differentiated [4]. Again, while LCI is reported to help highlight superficial non-ampullary duodenal epithelial tumors (SNADETs), its usefulness in the differential diagnosis of duodenal adenoma and adenocarcinoma remains insufficiently documented [5,6]. We present a case of duodenal adenocarcinoma, where LCI proved useful in its preoperative diagnosis, along with associated endoscopic and pathological findings of the lesion.

## Case presentation

A 67-year-old man was referred to our hospital for a positive fecal occult blood test and mild anemia (Hb, 10.1g/dL). He had a history of hypertension, hyperlipidemia, hyperuricemia, and atrial fibrillation, and had been treated with edoxaban. He also had a family history of colorectal cancer (his father) and angina pectoris (his mother). Colonoscopy findings only included minor adenomas.

**Figure 1:**
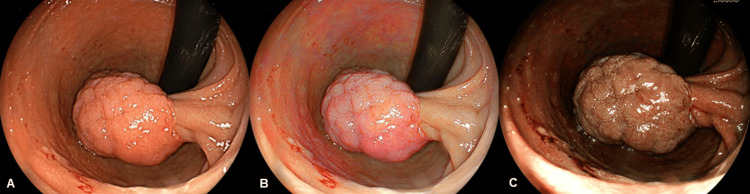
Esophagogastroduodenoscopy (EGD). (A) 20-mm whitish, partially reddish, pedunculated lesion located in the duodenal bulb on white light imaging; (B) The lesion was depicted as a whitish, pedunculated lesion with its central and inferior areas shown in orangish and reddish colors, respectively, during linked color imaging; (C) It was seen as a brownish, pedunculated lesion during blue laser imaging.

Esophagogastroduodenoscopy (EGD) revealed a 20-mm-sized, whitish, partially reddish, pedunculated lesion located in the duodenal bulb on white light imaging (WLI) (Figure 1A). The lesion was depicted as a whitish, pedunculated lesion with its central and inferior areas shown as orangish and reddish, respectively, during LCI (Figure 1B) and as a brownish, pedunculated lesion on blue laser imaging (BLI) (Figure 1C).

Based on these combined findings, the lesion was diagnosed as an early duodenal adenocarcinoma, despite its EGD biopsy-based diagnosis as an adenoma. Endoscopic mucosal resection (EMR) was performed on the suspected adenocarcinoma, which was thought to be the cause of anemia, for biopsy and endoscopic diagnosis. No adverse events such as bleeding or perforation associated with EMR were observed.

**Figure 2:**
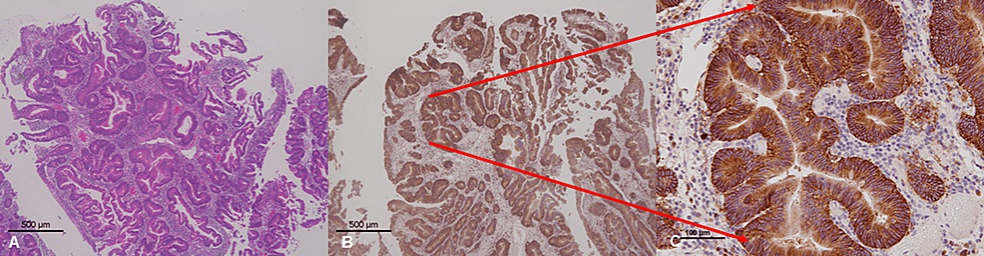
Pathological findings. (A) The lesion was confirmed in histological examination as an adenocarcinoma contained within an adenoma: papillary, type 0-Ip, measuring 20x20 mm, pTis (M), involving no lymphovascular invasion; (B, C) Immunohistological staining identified an adenocarcinomatous component positive for carbonic anhydrase IX (CA9).

Indeed, the lesion was revealed in the histological examination as an adenocarcinoma contained within an adenoma: papillary, type 0-Ip, measuring 20x20 mm, pTis (M), involving no lymphovascular invasion (Figure 2A). Furthermore, immunohistological staining identified an adenocarcinomatous component positive for carbonic anhydrase IX (CA9) (Figures 2B, 2C).

## Discussion

Our case has a few clinical implications that could potentially affect the differential diagnosis of adenocarcinoma versus adenoma. To begin with, LCI may prove effective in the differential diagnosis of adenocarcinoma by highlighting the tumor mucosa as orangish or reddish as well as in the diagnosis of SNADETs.

Duodenal high-grade adenoma or carcinoma has been endoscopically characterized as a lesion measuring ≥ 20 mm or ≥ 10 mm in diameter and involving erythema (i.e., reddish color) and a nodular rough surface [7-9]. LCI has recently been reported to assist in the diagnosis of SNADETs, with Okimoto et al. reporting that LCI may improve the visibility of SNADETs by highlighting these lesions as orange or orange-reddish [5]. Furthermore, Kitae et al. demonstrated that adenocarcinoma is significantly more frequently associated with the presence of reddish and orange colors (both on LCI), as well as lobulation, depression, and marginally white opaqueness, than adenoma [6].

In the present case, the lesion measured 20 mm in diameter with its nodularity shown to be uniform, which made it difficult to establish its differential diagnosis. On the other hand, LCI highlighted the central and inferior areas of the lesion in orangish and reddish colors, thus commending its use in the endoscopic diagnosis of duodenal adenocarcinoma.

Also of note in the present case was that CA9 may prove a viable prognostic marker for duodenal adenocarcinoma as a stable transmembrane zinc metalloenzyme shown to play a key role in tumor metabolism [10,11]. In studies of CA9-expressing duodenal adenocarcinomas published to date [12,13], CA9 expression was reported to be significantly correlated with better tumor differentiation, no lymph node metastasis, tumor downgrading, and no perineural and lymphatic invasion [12], while CA9 overexpression tended to be associated with no regional lymph node metastasis [13].

## Conclusions

In the present case, duodenal adenocarcinoma was effectively highlighted by LCI and confirmed by pathological examination as an adenocarcinoma contained within an adenoma: a papillary, localized lesion with no lymphovascular invasion. This case appears to underpin the usefulness of LCI in the differential diagnosis of duodenal adenocarcinoma versus adenoma. The endoscopic and pathological images given in the report should offer a clear demonstration of the case made, while further investigation is required in more patients with suspected duodenal adenocarcinoma.
